# Supplementary material for: Systematic analysis of human colorectal cancer scRNA-seq revealed limited pro-tumoral IL-17 production potential in gamma delta T cells
Source: Neoplasia. 2024 Oct 24;58:101072. doi: 10.1016/j.neo.2024.101072 (PMC11539345; doi:10.1016/j.neo.2024.101072)
Supplement: Supplementary file 1 [file mmc1.pdf]

Figure S1

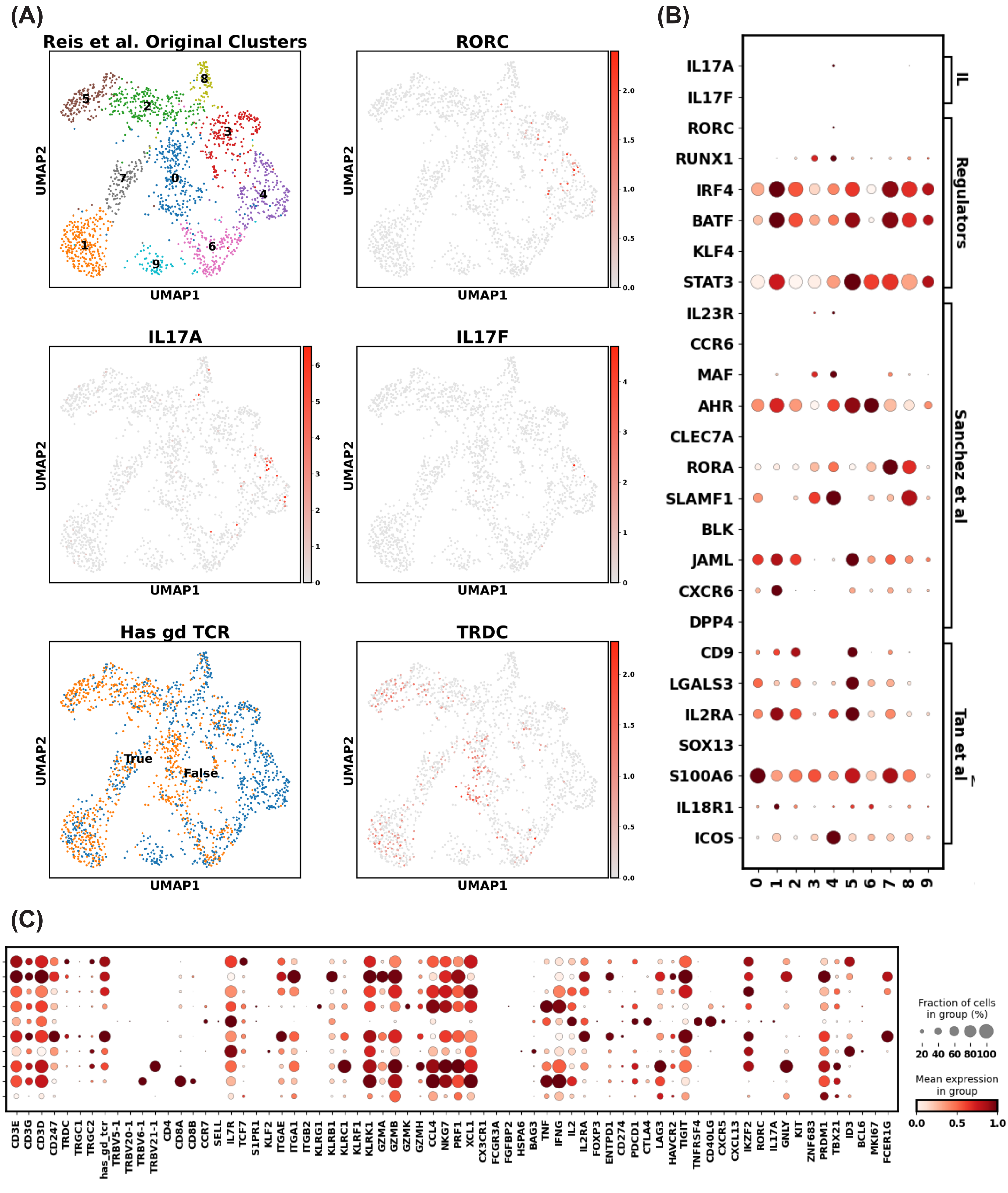

(A)
Reis et al. Original Clusters
RORC
IL17A
IL17F
Has gd TCR
TRDC
UMAP1
UMAP2
True
False
(B)
IL17A
IL17F
RORC
RUNX1
IRF4
BATF
KLF4
STAT3
IL23R
CCR6
MAF
AHR
CLEC7A
RORA
SLAMF1
BLK
JAML
CXCR6
DPP4
CD9
LGALS3
IL2RA
SOX13
S100A6
IL18R1
ICOS
IL
Regulators
Sanchez et al
Tan et al
(C)
CD3E
CD3G
CD3D
CD247
TRDC
TRGC1
TRGC2
has_gd_tcr
TRBV5-1
TRBV20-1
TRBV6-1
TRBV21-1
CD4
CD8A
CD8B
CCR7
SELL
IL7R
TCF7
S1PR1
KLF2
ITGAE
ITGA1
ITGB2
KLRG1
KLRB1
KLRC1
KLRF1
KLRK1
GZMA
GZMB
GZMK
GZMH
CCL4
NKG7
PRF1
XCL1
CX3CR1
FCGR3A
FGFBP2
HSPA6
BAG3
TNF
IFNG
IL2
IL2RA
FOXP3
ENTPD1
CD274
PDCD1
CTLA4
LAG3
HAVCR2
TIGIT
TNFRSF4
CD40LG
CXCR5
CXCL13
IKZF2
RORC
IL17A
GNLY
KIT
ZNF683
PRDM1
TBX21
ID3
BCL6
MKI67
FCER1G
Fraction of cells in group (%)
20 40 60 80 100
Mean expression in group
0.0 0.5 1.0

Figure S2

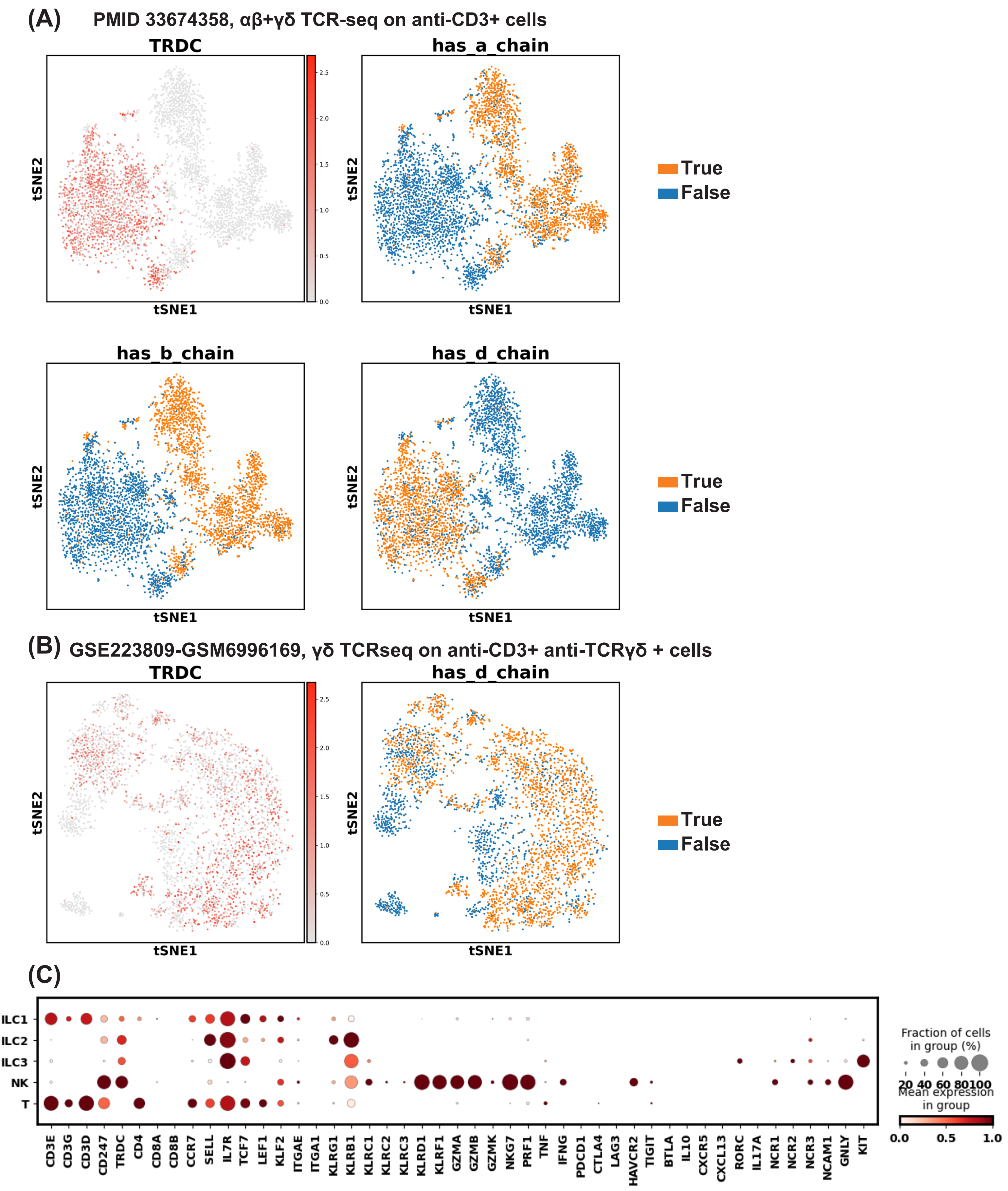

(A)
PMID 33674358, αβ+γδ TCR-seq on anti-CD3+ cells
TRDC
has_a_chain
has_b_chain
has_d_chain
tSNE1
tSNE2
True
False
(B)
GSE223809-GSM6996169, γδ TCRseq on anti-CD3+ anti-TCRγδ + cells
TRDC
has_d_chain
(C)
ILC1
ILC2
ILC3
NK
T
CD3E
CD3G
CD3D
CD247
TRDC
CD4
CD8A
CD8B
CCR7
SELL
IL7R
TCF7
LEF1
KLF2
ITGAE
ITGA1
KLRG1
KLRB1
KLRC1
KLRC2
KLRC3
KLRD1
KLRF1
GZMA
GZMB
GZMK
NKG7
PRF1
TNF
IFNG
PDCD1
CTLA4
LAG3
HAVCR2
TIGIT
BTLA
IL10
CXCR5
CXCL13
RORC
IL17A
NCR1
NCR2
NCR3
NCAM1
GNLY
KIT
Fraction of cells in group (%)
20 40 60 80 100
Mean expression in group
0.0 0.5 1.0

Figure S3

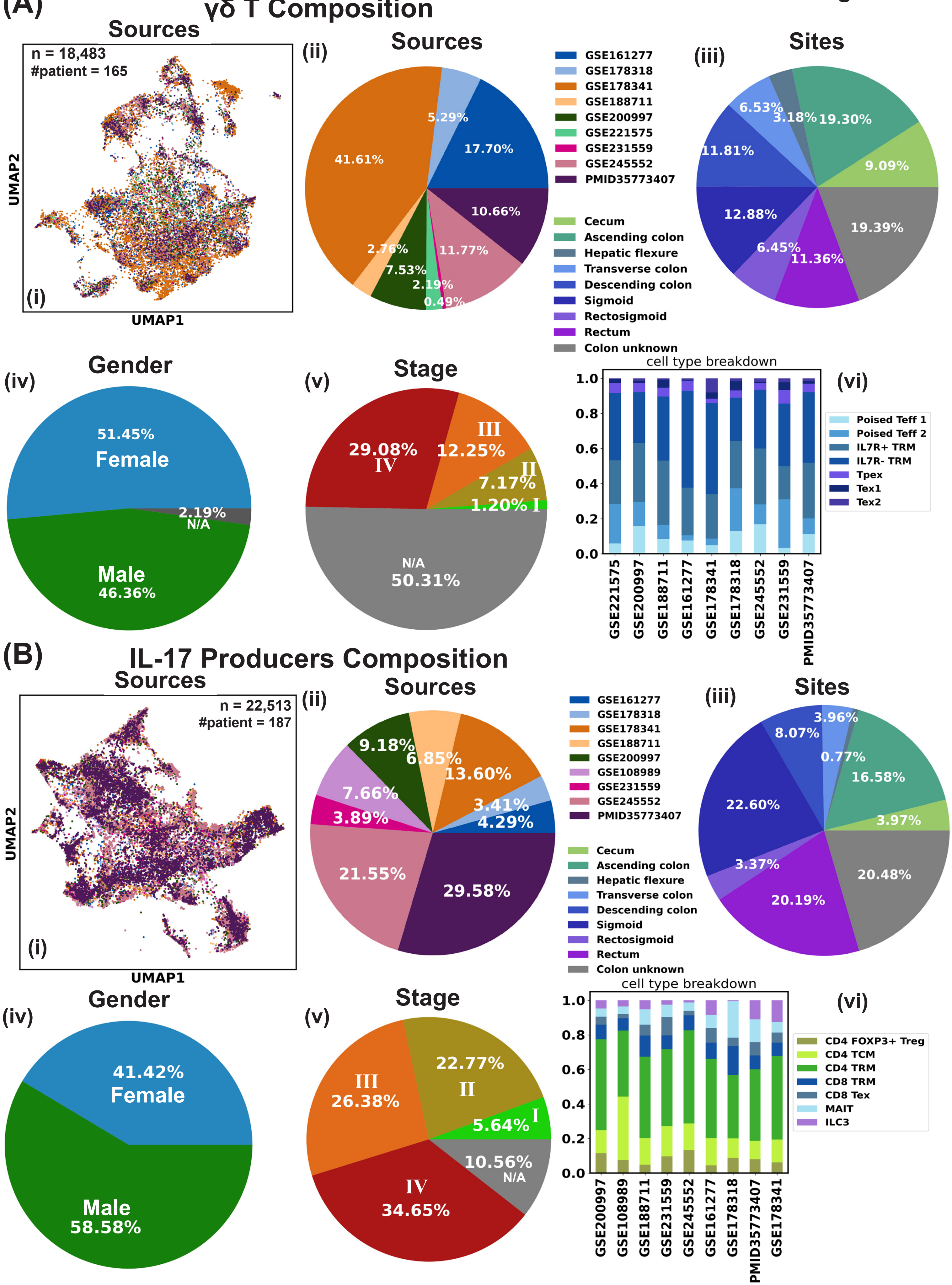

(A)
γδ T Composition
Sources
n = 18,483
#patient = 165
UMAP2
UMAP1
(i)
(ii)
Sources
GSE161277
GSE178318
GSE178341
GSE188711
GSE200997
GSE221575
GSE231559
GSE245552
PMID35773407
5.29%
17.70%
41.61%
10.66%
2.76%
11.77%
7.53%
2.19%
0.49%
(iii)
Sites
6.53%
3.18%
19.30%
11.81%
9.09%
12.88%
19.39%
6.45%
11.36%
Cecum
Ascending colon
Hepatic flexure
Transverse colon
Descending colon
Sigmoid
Rectosigmoid
Rectum
Colon unknown
(iv)
Gender
51.45%
Female
2.19%
N/A
Male
46.36%
(v)
Stage
29.08%
IV
III
12.25%
II
7.17%
1.20% I
N/A
50.31%
cell type breakdown
(vi)
Poised Teff 1
Poised Teff 2
IL7R+ TRM
IL7R- TRM
Tpex
Tex1
Tex2
GSE221575
GSE200997
GSE188711
GSE161277
GSE178341
GSE178318
GSE245552
GSE231559
PMID35773407
(B)
IL-17 Producers Composition
Sources
n = 22,513
#patient = 187
UMAP2
UMAP1
(i)
(ii)
Sources
GSE161277
GSE178318
GSE178341
GSE188711
GSE200997
GSE108989
GSE231559
GSE245552
PMID35773407
9.18%
6.85%
13.60%
7.66%
3.41%
3.89%
4.29%
21.55%
29.58%
(iii)
Sites
3.96%
8.07%
0.77%
16.58%
22.60%
3.97%
3.37%
20.48%
20.19%
Cecum
Ascending colon
Hepatic flexure
Transverse colon
Descending colon
Sigmoid
Rectosigmoid
Rectum
Colon unknown
(iv)
Gender
41.42%
Female
Male
58.58%
(v)
Stage
22.77%
III
II
26.38%
5.64% I
10.56%
N/A
IV
34.65%
cell type breakdown
(vi)
CD4 FOXP3+ Treg
CD4 TCM
CD4 TRM
CD8 TRM
CD8 Tex
MAIT
ILC3
GSE200997
GSE108989
GSE188711
GSE231559
GSE245552
GSE161277
GSE178318
PMID35773407
GSE178341

Figure S4

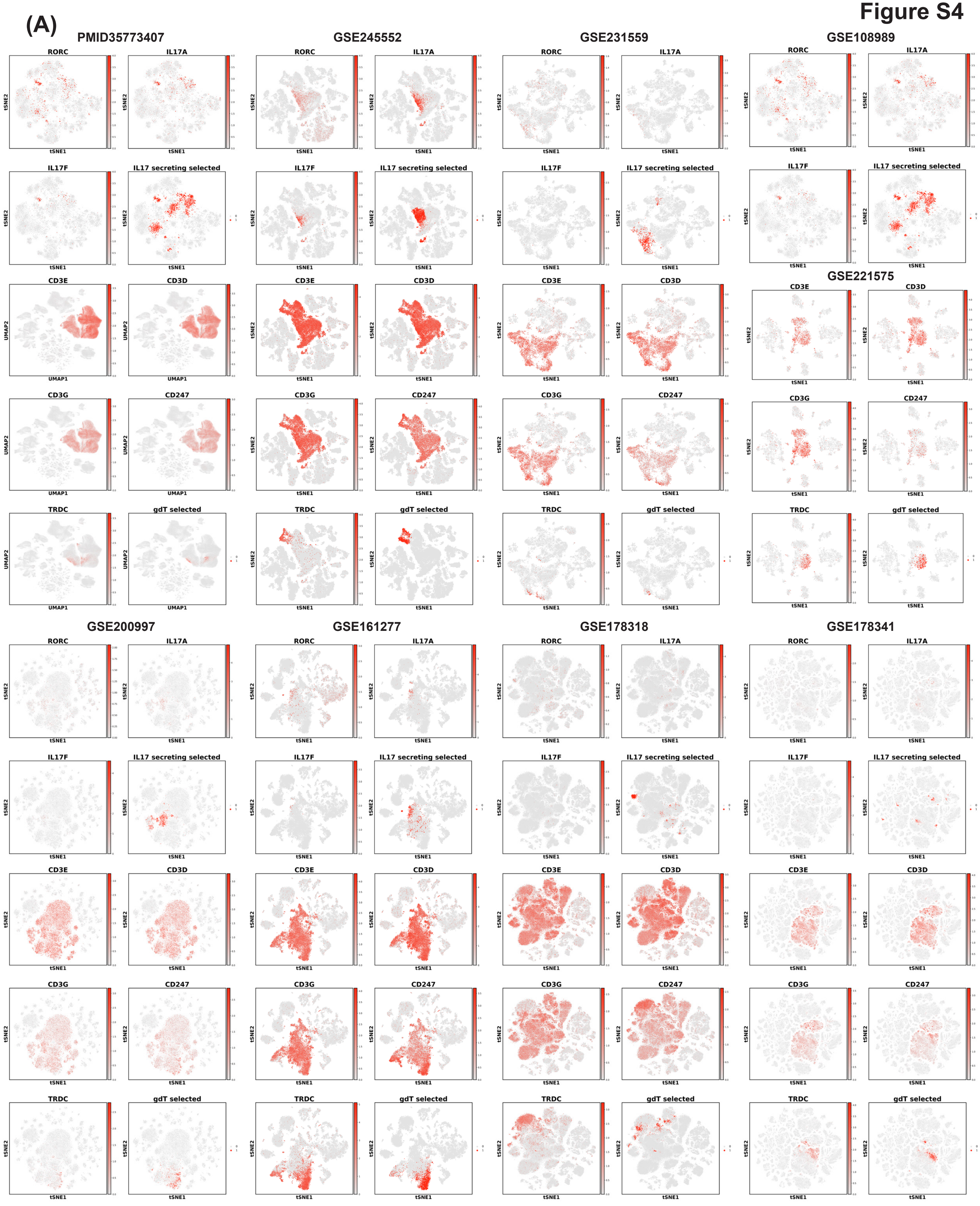
(A)
PMID35773407
GSE245552
GSE231559
GSE108989
GSE221575
GSE200997
GSE161277
GSE178318
GSE178341
RORC
IL17A
IL17F
IL17 secreting selected
CD3E
CD3D
CD3G
CD247
TRDC
gdT selected
tSNE1
tSNE2
UMAP1
UMAP2

Figure S4

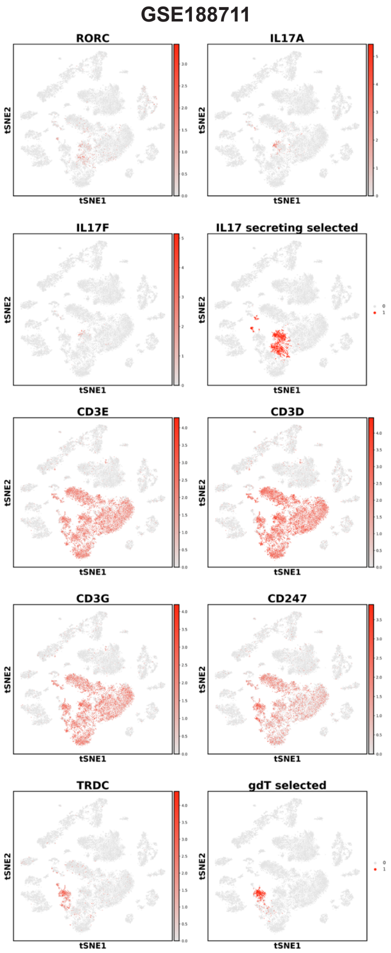
GSE188711
RORC
IL17A
IL17F
IL17 secreting selected
CD3E
CD3D
CD3G
CD247
TRDC
gdT selected
tSNE1
tSNE2

Figure S5

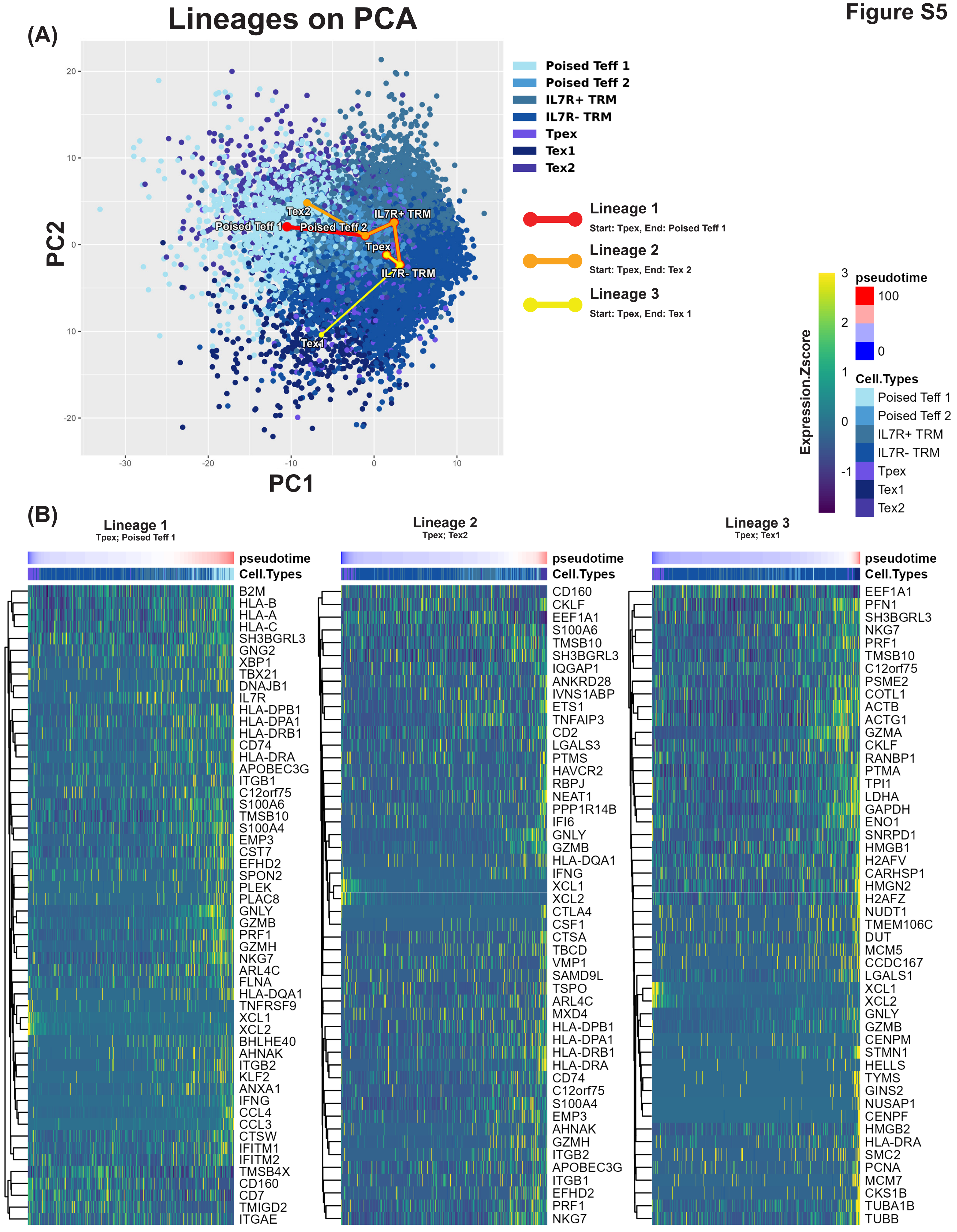
(A)
Lineages on PCA
PC1
PC2
Poised Teff 1
Poised Teff 2
IL7R+ TRM
IL7R- TRM
Tpex
Tex1
Tex2
Lineage 1
Start: Tpex, End: Poised Teff 1
Lineage 2
Start: Tpex, End: Tex 2
Lineage 3
Start: Tpex, End: Tex 1
Expression.Zscore
pseudotime
100
0
Cell.Types
Poised Teff 1
Poised Teff 2
IL7R+ TRM
IL7R- TRM
Tpex
Tex1
Tex2
(B)
Lineage 1
Tpex; Poised Teff 1
pseudotime
Cell.Types
B2M HLA-B HLA-A HLA-C SH3BGRL3 GNG2 XBP1 TBX21 DNAJB1 IL7R HLA-DPB1 HLA-DPA1 HLA-DRB1 CD74 HLA-DRA APOBEC3G ITGB1 C12orf75 S100A6 TMSB10 S100A4 EMP3 CST7 EFHD2 SPON2 PLEK PLAC8 GNLY GZMB PRF1 GZMH NKG7 ARL4C FLNA HLA-DQA1 TNFRSF9 XCL1 XCL2 BHLHE40 AHNAK ITGB2 KLF2 ANXA1 IFNG CCL4 CCL3 CTSW IFITM1 IFITM2 TMSB4X CD160 CD7 TMIGD2 ITGAE
Lineage 2
Tpex; Tex2
pseudotime
Cell.Types
CD160 CKLF EEF1A1 S100A6 TMSB10 SH3BGRL3 IQGAP1 ANKRD28 IVNS1ABP ETS1 TNFAIP3 CD2 LGALS3 PTMS HAVCR2 RBPJ NEAT1 PPP1R14B IFI6 GNLY GZMB HLA-DQA1 IFNG XCL1 XCL2 CTLA4 CSF1 CTSA TBCD VMP1 SAMD9L TSPO ARL4C MXD4 HLA-DPB1 HLA-DPA1 HLA-DRB1 HLA-DRA CD74 C12orf75 S100A4 EMP3 AHNAK GZMH ITGB2 APOBEC3G ITGB1 EFHD2 PRF1 NKG7
Lineage 3
Tpex; Tex1
pseudotime
Cell.Types
EEF1A1 PFN1 SH3BGRL3 NKG7 PRF1 TMSB10 C12orf75 PSME2 COTL1 ACTB ACTG1 GZMA CKLF RANBP1 PTMA TPI1 LDHA GAPDH ENO1 SNRPD1 HMGB1 H2AFV CARHSP1 HMGN2 H2AFZ NUDT1 TMEM106C DUT MCM5 CCDC167 LGALS1 XCL1 XCL2 GNLY GZMB CENPM STMN1 HELLS TYMS GINS2 NUSAP1 CENPF HMGB2 HLA-DRA SMC2 PCNA MCM7 CKS1B TUBA1B TUBB
